# Quantitative proteomics reveals Piccolo as a candidate serological correlate of recovery from Guillain-Barré syndrome

**DOI:** 10.18632/oncotarget.12789

**Published:** 2016-10-20

**Authors:** Lourdes Mateos-Hernández, Margarita Villar, Ernesto Doncel-Pérez, Marco Trevisan-Herraz, Ángel García-Forcada, Francisco Romero Ganuza, Jesús Vázquez, José de la Fuente

**Affiliations:** ^1^ SaBio. Instituto de Investigación en Recursos Cinegéticos IREC-CSIC-UCLM-JCCM, Ronda de Toledo s/n, 13005 Ciudad Real, Spain; ^2^ Hospital Nacional de Parapléjicos, Servicio de Salud de Castilla La Mancha (SESCAM), Finca La Peraleda s/n, 45071 Toledo, Spain; ^3^ Centro Nacional de Investigaciones Cardiovasculares, Madrid, Spain; ^4^ Department of Veterinary Pathobiology, Center for Veterinary Health Sciences, Oklahoma State University, Stillwater, OK, USA

**Keywords:** immunity, neuropathy, proteomics, biomarker, Guillain-Barré, neurology, Immunology and Microbiology Section, Immune response, Immunity

## Abstract

Guillain-Barré syndrome (GBS) is an autoimmune-mediated peripheral neuropathy of unknown cause. However, about a quarter of GBS patients have suffered a recent bacterial or viral infection, and axonal forms of the disease are especially common in these patients. Proteomics is a good methodological approach for the discovery of disease biomarkers. Until recently, most proteomics studies of GBS and other neurodegenerative diseases have focused on the analysis of the cerebrospinal fluid (CSF). However, serum represents an attractive alternative to CSF because it is easier to sample and has potential for biomarker discovery. The goal of this research was the identification of serum biomarkers associated with recovery from GBS. To address this objective, a quantitative proteomics approach was used to characterize differences in the serum proteome between a GBS patient and her healthy identical twin in order to lessen variations due to differences in genetic background, and with additional serum samples collected from unrelated GBS (*N* = 3) and Spinal Cord Injury (SCI) (*N* = 3) patients with similar medications. Proteomics results were then validated by ELISA using sera from additional GBS patients (*N* = 5) and healthy individuals (*N* = 3). All GBS and SCI patients were recovering from the acute phase of the disease. The results showed that Piccolo, a protein that is essential in the maintenance of active zone structure, constitutes a potential serological correlate of recovery from GBS. These results provided the first evidence for the Piccolo's putative role in GBS, suggesting a candidate target for developing a serological marker of disease recovery.

## INTRODUCTION

Guillain-Barré syndrome (GBS) is an immune-mediated peripheral neuropathy that has been identified as the main cause of the acute neuromuscular paralysis, with an annual incidence ranging from 0.81 to 1.89 cases per 100.000 people [1, 2]. GBS is characterized by rapidly evolving ascending weakness, mild sensory loss and hyporeflexia or areflexia, progressing to a nadir over up to four weeks [2]. Besides the classic presentation of ascending paralysis in demyelinating GBS, different subtypes of GBS such as Acute Inflammatory Demyelinating Polyneuropathy (AIDP), Miller Fisher Syndrome (MFS), Acute Motor Axonal Neuropathy (AMAN), Acute Motor Sensory Axonal Neuropathy (AMSAN), Acute Panautonomic Neuropathy and Bickerstaff's Brainstem Encephalitis (BBE) have been described [1–4].

Although GBS is considered to be an autoimmune disease with the involvement of both cellular and humoral immune responses [2], the precise cause of the syndrome is unknown [2, 5–9]. About a quarter of patients with GBS have suffered a recent bacterial or viral infection, and axonal forms of the disease are especially common in these patients [2, 10–14]. Recent results showed that infection leads to antibody production, which cross-reacts with gangliosides and other glycolipids leading to myelin destruction by complement activation or by antibodies targeting macrophages via the Fc receptor and leading to both demyelination and nerve conduction failure [2, 14]. Furthermore, recent results point at pathogen-derived proteins as triggers of molecular mimicry involved in the GBS [15–17].

The post-genomic or “omics” technologies allow a high throughput analysis of cell, tissue or organism response to different conditions, thus improving our understanding of the biological processes involved and the possibilities for the identification of targets for prognosis, therapeutic and preventive interventions. However, few studies have applied “omics” technologies to the study of GBS. Recently, we used a transcriptomics approach for the identification of biomarkers associated with recovery from GBS [18]. The results showed the presence of up- and down-regulated transcripts in response to GBS in these patients when compared to matching healthy control individuals. These results identified new biomarkers associated with GBS recovery and suggested that early growth response gene-2 (EGR2) over-expression has a pivotal role in the down-regulation of cytokines implicated in the pathophysiology of this acute neuropathy [18]. At the protein level, few studies have applied proteomics for the characterization of the cerebrospinal fluid (CSF) proteome in GBS patients (see for example [19–21]). However, the serum proteome has been only partially characterized in patients with GBS and could be a source of potential biomarkers for this disease [22].

Treatment strategies for patients with GBS include plasmapheresis, intravenous immunoglobulin administration and immunosuppressive drugs [2, 4]. However, new diagnosis and treatment strategies are necessary to prevent both the development of the syndrome as well as the persistent disability in GBS patients [4]. The results of previous studies showed the possibilities of “omics” technologies for the study of GBS [15–19]. A better understanding of the immunological and pathological mechanisms involved in GBS is essential for the development of new treatments. Additionally, molecular studies of GBS using high throughput technologies such as proteomics may result in the identification of new predictors and disease biomarkers.

The goal of the research reported here was the identification of potential serological biomarkers associated with the GBS. To address this objective, a quantitative proteomics approach was used to characterize differences in the serum proteome between GBS patients, unrelated Spinal Cord Injury (SCI) patients with similar medications and healthy individuals. The results suggested that Piccolo might be a serological correlate of recovery from GBS.

## RESULTS AND DISCUSSION

### Serum proteome in GBS patients reveals Piccolo protein as a candidate serological correlate of recovery from GBS

Until recently, most proteomics studies of GBS and other neurodegenerative diseases such as multiple sclerosis have focused on the analysis of the CSF [19–21, 23]. However, while the CSF might be considered more tissue specific, patients sample collection is invasive and poses risks that range from headache to nerve damage and paraplegia, therefore resulting unacceptable for regular sampling [24]. Serum represents an attractive alternative to CSF because it is easier to sample and has potential for biomarker discovery [22, 25]. Although serum lacks tissue specificity and reflects the protein content of various tissues and cell types, serum proteomics studies should be successful if based on groups of well-phenotyped patients [23–25].

**Figure 1:**
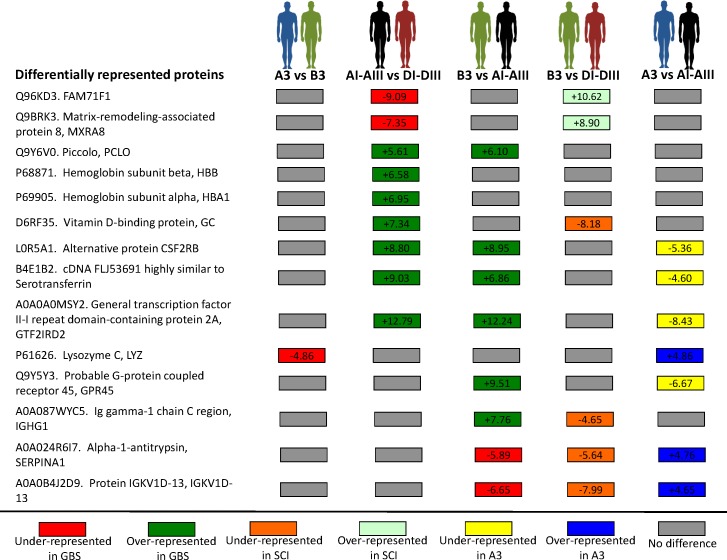
Differentially represented serum proteins Serum samples collected from a GBS patient (A3) and her healthy identical twin (B3) [18], and from unrelated GBS (*N* = 3; AI-AIII) and SCI (*N* = 3; DI-DIII) patients with similar medications were included in the proteomics analysis (Table 1). The 5% FDR was used as criterion for peptide identification. Protein identification (Uniprot accession) and name are shown. For over-represented and under-represented protein quantifications, a standardized variable of protein fold changes was used (Zq), where statistical significance of protein abundance changes was considered using a 1% FDR.

Our study was based on well-defined GBS and unrelated SCI patients on similar medications, and healthy control individuals [18]. Data on patients and control individuals were described in detail in [18] and summarized in Table 1. The proteomics analysis was conducted with serum samples collected from a GBS patient (A3) and her healthy identical twin (B3) in order to reduce variations due to differences in genetic background [18], and with additional serum samples collected from unrelated GBS (*N* = 3; AI-AIII) and SCI (*N* = 3; DI-DIII) patients with similar medications (Table 1). Serum samples were collected simultaneously from the patient and her control healthy twin at conclusion of the locomotion rehabilitation program when the patient was close to be discharged from the hospital, and when differences at the transcriptomics level were identified between the GBS patient and her healthy identical twin [18]. The remaining GBS and SCI patients were also recovering from the acute phase of the disease (Table 1). Pairwise comparisons were done between iTRAQ proteomics data from all samples to select differentially represented proteins selected with 1% FDR (Figure 1).

**Table 1:** Summary of GBS and SCI patients, and healthy individuals included in the study

Identification	Individual	Clinical diagnosis	Age	Gender	Functional status
A3	GBS patient twin	GBS, AMSAN variant	23	F	Able to stand and walk with help
AI	GBS patient I	GBS, AMAN variant	31	M	Generalized muscle atrophy of 4 limbs, able to stand, wheelchair-bound for displacements, dysphagia, dysarthria
AII	GBS patient II	GBS, Undetermined variant	84	F	Wheelchair-bound, lack of voluntary movements on 4 limbs
AIII	GBS patient III	GBS, AMAN variant	68	M	Able to stand and walk short distances with help, wheelchair for long displacements
AIV	GBS patient IV	GBS, AMAN variant	70	M	Able to perform daily basic activities with help, stands and walks with gaitaid walker device
AV	GBS patient V	GBS, AMAN variant	54	M	Wheelchair bound for displacements, able to stand short periods, with help
AVI	GBS patient VI	GBS, AMAN variant	71	F	Mild tetraparesia, able to stand and walk short distances with help, able to perform basic daily activities with help
AVII	GBS patient VII	GBS, Undetermined variant	55	F	Able to perform basic daily activities, stands and walks with gaitaid walker device
DI	SCI patient I	SCI at cervical level 7	64	M	Complete paraplegia, wheel chair for any displacement
DII	SCI patient II	SCI at cervical level 4	56	M	Tetraplegic, wheelchair-bound
DIII	SCI patient III	SCI at cervical level 4	44	M	Tetraplegic, wheelchair-bound
DIV	SCI patient IV	SCI at cervical level 5	51	F	Incomplete tetraplegia, able to stand with help, wheelchair-bound fordisplacements
B3	Control healthy twin	Healthy	23	F	Healthy
CI	Control healthy individual I	Healthy	30	F	Healthy
CII	Control healthy individual II	Healthy	41	F	Healthy
CIII	Control healthy individual III	Healthy	55	M	Healthy

GBS patients (*N* = 8; A3, AI-AVII), SCI unrelated patients (*N* = 4; DI-DIV), and healthy individuals (*N* = 4; B3, Ci-CIII) were included in the study. GBS patients were sampled after recovering from the acute disease phase with similar medications. Medications are described in [18]. Abbreviations: GBS, Guillain-Barré syndrome; SCI, Spinal Cord Injury; AMSAN, Acute Motor Sensory Axonal Neuropathy; AMAN, Acute Motor Axonal Neuropathy; F, female; M, male.

**Figure 2:**
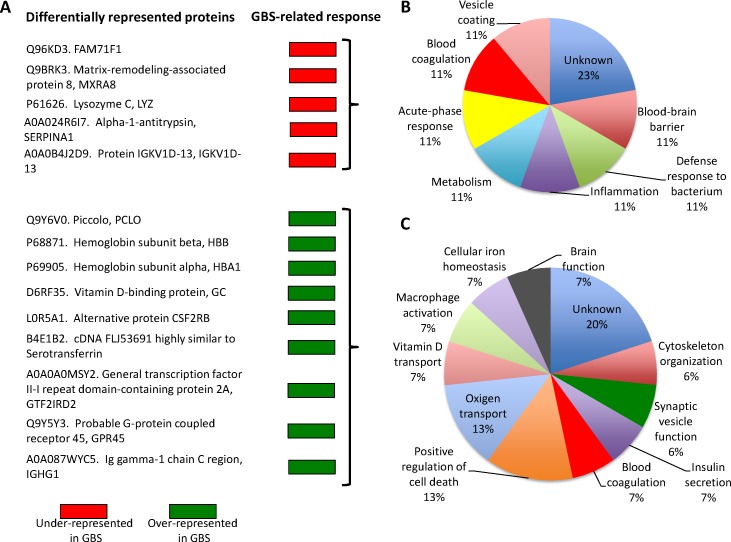
GBS-related serological response **A.** Differentially represented proteins were grouped as under-represented and over-represented in GBS patients when compared to SCI patients or healthy twin. Protein ontology analysis for biological process was done using the Blast2GO software (www.blast2go.com) for **B.** under-represented and **C.** over-represented proteins.

A total of 330 proteins were quantified in all samples (Supplemental Table S1), and of them 14 were differentially represented after pairwise comparisons between different groups (Figure 1 and Supplemental Table S1). The GBS-related response showed the differential representation of secreted proteins included in biological processes involved in GBS and other neuropathies, therefore suggesting their role in disease progression and recovery [16, 26–32] (Figure 2A-2C). However, the only protein that was differentially represented in GBS patients when compared to SCI patients (AI-AIII *vs*. DI-DIII) and healthy individual (B3 *vs*. AI-AIII), and not affected by comparison between GBS patients (A3 *vs*. AI-AIII), SCI patients and healthy individual (B3 *vs*. DI-DIII), or between identical twins (A3 *vs*. B3, which may reflect A3-specific response) was Piccolo (Figure 1). The fact that Piccolo serum protein levels did not differ between GBS (A3) and healthy (B3) identical twins or between A3 and GBS patients (AI-AIII), but differed between B3 and GBS patients (AI-AIII) precluded the existence of an A3-individual rather than a GBS group-associated response, which may be possible to identify in individuals with identical genetic background. Therefore, the Piccolo protein that was over-represented in GBS patients recovering from the acute disease phase (Figure 2A), was selected as a potential serological correlate of recovery from GBS.

### Validation of proteomics results confirm the potential of Piccolo as a serological correlate of recovery from GBS

**Figure 3:**
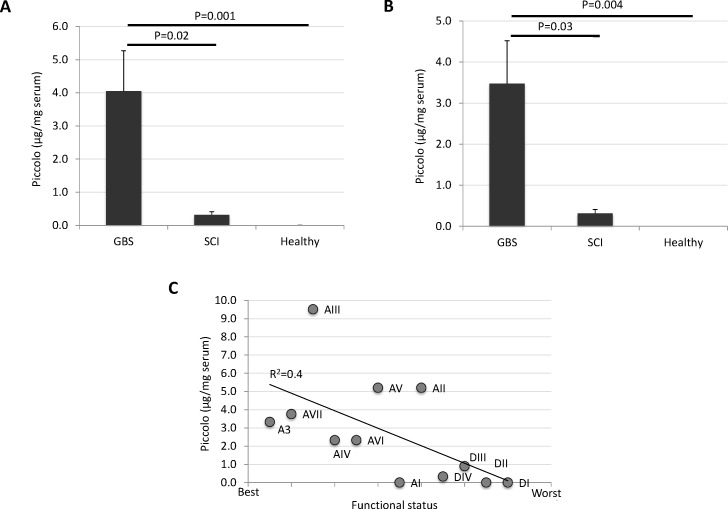
Piccolo as a potential serological correlate of recovery from GBS Sera from GBS (*N* = 8; A3, AI-AVII) and SCI (*N* = 4; DI-DIV) unrelated patients with similar medications, and healthy control individuals (*N* = 4; B3, CI-CIII), including the GBS patient (A3) and her healthy identical twin (B3) were included in the ELISA (Table 1). ELISA plates were coated with 100 μl/well of albumin-depleted sera at concentration of 2 ng/μl (0.2 μg serum/well). Mouse monoclonal anti-Piccolo antibodies were added at 1:1000 dilution and detected using anti-mouse IgG (μ-chain specific)-peroxidase antibodies produced in goat. Color was developed by the addition of TMB to measure the OD_450 nm_. For Piccolo protein quantitation, the human recombinant protein was used. Two technical replicates were included for each sample. The Piccolo concentration in serum samples was compared between GBS patients, SCI patients and healthy individuals by Student's t-test with unequal variance (*P* = 0.05). **A.** Piccolo serum levels in GBS (*N* = 8; A3, AI-AVII) patients, SCI (*N* = 4; DI-DIV) unrelated patients, and healthy control individuals (N = 4; B3, CI-CIII). **B.** Piccolo serum levels determined in GBS patients (AIV-AVII) and healthy individuals (CI-CIII) not included in the proteomics analysis. **C.** Negative correlation between Piccolo serum levels and patient functional status (Table 1). Correlation coefficient (R^2^) is shown.

For validation of proteomics results, a Piccolo ELISA was performed using sera from GBS (*N* = 8; A3, AI-AVII) and SCI (*N* = 4; DI-DIV) unrelated patients on similar medications, and healthy control individuals (*N* = 4; B3, CI-CIII), including the GBS patient (A3) and her healthy identical twin (B3) (Table 1). The results of the ELISA corroborated the proteomics results by showing higher Piccolo protein concentration in sera from GBS patients when compared to SCI patients and healthy individuals (Figure 3A). These results were similar when performing the analysis only with GBS patients (AIV-AVII) and healthy individuals (CI-CIII) not included in the proteomics analysis (Figure 3B), therefore providing support with an independent set of samples for the potential of Piccolo as a serological correlate of recovery from GBS. Additionally, a negative correlation was obtained between Piccolo serum levels and patient functional status (Table 1), suggesting again an increase in Piccolo serum levels during disease recovery (Figure 3C).

Antibodies against single ganglioside species remain the most established serological marker of GBS [33]. Recently, ELISA for the detection of antibodies against combinations of gangliosides and ganglioside-complex antibodies have emerged as a new method for the diagnostic of certain GBS variants, but do not seem to greatly improve the diagnosis of GBS [33]. Therefore, new serum markers are needed for better GBS diagnosis. The results of our study confirmed the potential of Piccolo as a serological correlate of recovery from GBS, and supported the conduction of additional experiments to validate its application as a serum marker for GBS.

### Piccolo's putative role during GBS

**Figure 4:**
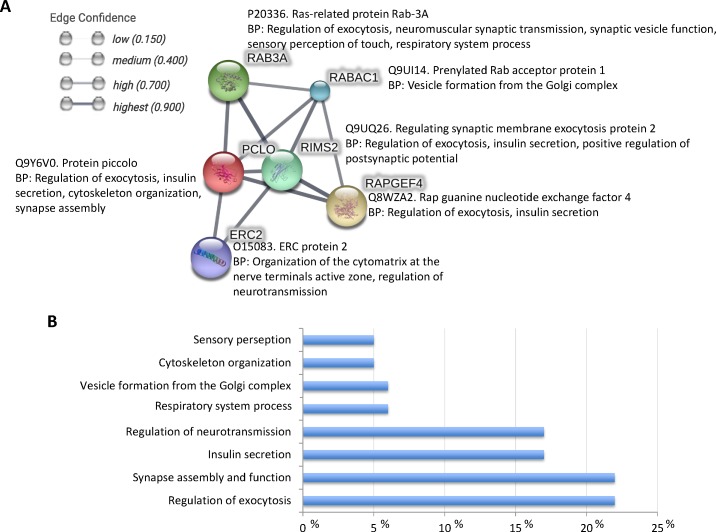
Piccolo-protein interactions **A.** STRING was used for the *in silico* characterization of Piccolo-protein interactions using a high confidence interaction score (0.700; http://bit.ly/2anoqgi). Each node represents all the proteins produced by a single protein-coding gene locus. Protein-protein associations represent proteins that jointly contribute to a shared function, but not necessarily physically bind to each other. **B.** Protein ontology analysis for biological process was done for Piccolo and interacting proteins using the Blast2GO software (www.blast2go.com).

Piccolo is a high molecular weight active zone specific scaffolding protein that is essential in the maintenance of active zone structure [34, 35]. This protein is involved in assembling presynaptic F-actin, gathering synaptic vesicles, and controlling synaptic transmission and voltage-gated calcium channel function [34, 35]. Piccolo is involved in multiple protein-protein interactions [35] and functional associations (Figure 4A). These interactions result in a role for Piccolo in multiple biological processes such as regulation of exocytosis, synapse assembly and function, insulin secretion, regulation of neurotransmission, cytoskeleton organization, and sensory perception (Figure 4B) affecting both central and peripheral nervous systems [35–37]. Additionally, Piccolo has been localized not only at cell junctions and cytoskeleton, but also in the extracellular exosome from human neural stem cells [38].

The presynaptic neuromuscular junction (NMJ) has been considered a potential target vulnerable to autoimmune attack in GBS and related diseases [2, 3, 10–16]. Therefore, Piccolo may be involved in recovering from GBS by reinstalling the activity of the presynaptic active zone [35, 39, 40], which helps restoring movement and function to a person's full potential [2, 3]. The increase in serum Piccolo levels observed in GBS patients during recovery from the acute phase of the disease may reflect protein secretion during molecular remodeling of the presynaptic active zone. This finding needs additional experiments using animal models (i.e. [39–41]) in order to characterize the role of Piccolo in the recovery from GBS, providing additional support for the results presented here.

In summary, using a quantitative serum proteomics approach we have shown that Piccolo constitutes a potential serological correlate of recovery from GBS. These results need to be validated using a larger number of GBS patients and animal models, but provided the first evidence for the Piccolo's putative role during GBS, suggesting a candidate target for developing a serological marker for disease recovery.

## MATERIALS AND METHODS

### Ethics statement

The use of human material, including peripheral blood serum samples from GBS patients, SCI patients and healthy individuals was approved by the Clinical Research Ethics Committee for Hospitals of Toledo City (permit No. 17), and informed consent was obtained from all individuals in compliance with the Helsinki Declaration. Blood samples of patients and controls were extracted by nursing personnel to patients and controls in the Paraplegics National Hospital (Toledo, Spain).

### Serum samples

For separation of serum from the total blood a sterile tube without anticoagulant was used. The blood from each individual (5 ml) was maintained in standing position at room temperature (RT) for clotting (20-30 min), and centrifuged at 1500 x g for 20 min at RT. Serum was collected and conserved at −20°C until used for cytokine protein analysis.

### Proteomics data acquisition and analysis

Serum samples collected from a GBS patient (A3) and her healthy identical twin (B3) [18], and from unrelated GBS (*N* = 3; AI-AIII) and SCI (*N* = 3; DI-DIII) patients with similar medications were included in the analysis (Table 1). The protein extracts (100 μg serum from each patient), were suspended in 50 μl of sample buffer and applied onto 1.2-cm wide wells of a conventional SDS-PAGE gel (0.75 mm-thick, 4% stacking, and 10% resolving). Then run was stopped as soon as the front entered 3 mm into the resolving gel, so that the whole proteome became concentrated in the stacking/resolving gel interface. The unseparated protein bands were visualized by Coomassie staining, excised, cut into cubes (2 mm^3^), and placed in 0.5 ml microcentrifuge tubes. The gel pieces were destained in acetonitrile:water (ACN:H_2_O, 1:1). Disulfide bonds from cysteinyl residues were reduced with 10 mM DTT for 1 h at 56°C, and then, thiol groups were alkylated with 50 mM iodoacetamide for 1 h at room temperature in darkness. Proteomes were finally digested *in situ* with sequencing grade trypsin (Promega, Madison, WI) as described by Shevchenko et al. [42] with minor modifications. The gel pieces were shrunk by removing all liquid using sufficient acetonitrile, which was pipetted out and the gel pieces were dried in a speed-vac. The dried gel pieces were re-swollen in 50 mM ammonium bicarbonate pH 8.8 with 60 ng/ml trypsin at 5:1 protein:trypsin (w/w) ratio. The tubes were kept in ice for 2 h and incubated at 37°C for 12 h. Digestion was stopped by the addition of 1% TFA. Whole supernatants were dried down and then desalted onto OASIS HLB Extraction Cartridges (Waters Corporation, Mildford, MA, USA) until the iTRAQ labeling. The resultant peptide mixture from proteins tryptic digest was labeled using chemicals from the iTRAQ reagent 8plex Multi-plex kit (reagents 113, 114, 115, 116, 117, 118, 119 and 121) (Applied Biosystems, Foster City, CA, USA) essentially as described by Köcher et al. [43]. Briefly, peptides were dissolved in 0.5 M triethylammonium bicarbonate (TEAB), adjusted to pH 8. For labeling, each iTRAQ reagent was dissolved in 50 *μ*L of isopropanol and added to the respective peptide mixture and then incubated at room temperature for two hours. Labelling was stopped by the addition of 0.1% formic acid. Whole supernatants were dried down and the eight samples were mixed to obtain the “8plex-labeled mixture”. The mixture was desalted onto OASIS HLB Extraction Cartridges (Waters Corporation) until the mass spectrometric analysis. The desalted 8plex-labeled mixture was dried, resuspended in 10 l of 0.1% formic acid and analyzed by reverse phase liquid chromatography coupled to mass spectrometry (RP-LC-MS/MS) in an Easy-nLC II system coupled to an ion trap LTQ-Orbitrap-Velos-Pro mass spectrometer (Thermo Scientific). The peptides were concentrated (on-line) by reverse phase chromatography using a 0.1mm × 20 mm C18 RP precolumn (Proxeon), and then separated using a 0.075mm x 250 mm C18 RP column (Proxeon) operating at 0.3 μl/min. Peptides were eluted using a 240-min dual gradient from 5 to 25% solvent B in 180 min followed by gradient from 25 to 40% solvent B over 240 min (Solvent A: 0,1% formic acid in water, solvent B: 0,1% formic acid, 80% acetonitrile in water). ESI ionization was done using a Nano-bore emitters Stainless Steel ID 30 μm (Proxeon) interface. The instrument method consisted of a data-dependent top-20 experiment with an Orbitrap MS1 scan at a resolution (*m*/Δ*m*) of 30,000 followed by either twenty high erenergy collision dissociation (HCD) MS/MS mass-analyzed in the Orbitrap at 7,500 (Δ*m*/*m*) resolution. MS2 experiments were performed using HCD to generate high resolution and high mass accuracy MS2 spectra. The minimum MS signal for triggering MS/MS was set to 500. The lock mass option was enabled for both MS and MS/MS mode and the polydimethylcyclosiloxane ions (protonated (Si(CH3)2O))6; *m*/*z* 445.120025) were used for internal recalibration of the mass spectra. Peptides were detected in survey scans from 400 to 1600 amu (1 μscan) using an isolation width of 2 u (in mass-to-charge ratio units), normalized collision energy of 40% for HCD fragmentation, and dynamic exclusion applied during periods of 30 seconds. Precursors of unknown or +1 charge state were rejected.

The raw files were analyzed using Thermo Proteome Discoverer 1.4, with a Uniprot database containing 147.854 entries of *Homo sapiens* (September 8, 2015). For database searching, parameters were selected as follows: trypsin digestion with 2 maximum missed cleavages, precursor mass tolerance 800 ppm, fragment mass tolerance of 0.05 Da, allowing variable modifications methionine oxidation, cysteine carbamidomethylation, and lysine oTRAQ 8-plex (of +304.205 Da), and fixed modification of peptide N-terminus for iTRAQ 8-plex. The same MSMS spectra were also searched against inverted databases constructed from the same target databases. Peptide identification from MSMS data was performed using the probability ratio method [44]. False discovery rates (FDR) of peptide identifications were calculated using the refined method [45, 46], and 5% FDR was used as criterion for peptide identification. Each peptide was assigned only to the best protein proposed by the Proteome Discoverer algorithm. Quantitative information was extracted from MS/MS spectra using a quantitative proteomics software package (QuiXoT) [47], and protein abundance changes were analyzed using the Generic Integration Algorithm [48]. Calculation of statistical weights of each quantitation at the spectrum level was performed according to the WSPP model [47]. The validity of the null hypothesis at each one of the levels (spectrum, peptide, and protein) was carefully checked by plotting the cumulative distributions of the standardized variables for each level (Zs, Zp and Zq), which were calculated as a function of the corresponding fold-changes (Xs, Xp and Xq) and the corresponding general variances (s^2^s, s^2^p and s^2^q). Statistical significance of outliers (at spectrum and peptide levels) and expression changes (at protein level) were considered using the calculated FDR values [47].

### Piccolo ELISA

Sera from GBS (*N* = 8; A3, AI-AVII) and SCI (*N* = 4; DI-DIV) unrelated patients with similar medications, and healthy control individuals (*N* = 4; B3, CI-CIII), including the GBS patient (A3) and her healthy identical twin (B3) were included in the analysis (Table 1). Sera were albumin depleted with Pierce albumin depletion Kit (Thermo Scientific, San Jose, CA, USA) following manufacturer's instructions. ELISA plates were coated with 100 μl/well of albumin-depleted sera at concentration of 2 ng/μl in carbonate/bicarbonate buffer and incubated overnight at 4°C. Then, 100 μl of blocking buffer (5% skim milk powder in phosphate buffered saline, PBS) were added to each well and incubated 1 hr at room temperature (RT) followed by five washes with PBS supplemented with 0.05% Tween 20 (PBST) (Sigma-Aldrich, Madrid, Spain). The mouse monoclonal anti-human Piccolo antibodies [6H9-B6] (ab101654; Abcam, Cambridge, UK), which are specific for human Piccolo and without cross-reactivity to Bassoon (http://www.abcam.com/piccolo-antibody-6h9-b6-ab101654-references.html) were added at 1:1000 dilution and incubated for 1 hr at 37°C followed by five washes with PBST. For the detection of Piccolo, 100 μl of anti-mouse IgG (μ-chain specific)-peroxidase antibodies produced in goat (Sigma-Aldrich) were added at a 1:1000 dilution in blocking buffer. Plates were incubated for 1h at RT, and subsequently washed with PBST five times. Color was developed by the addition of 100 μl of 3,3',5,5'-tetramethylbenzidine (TMB) (Promega Biotech, Madrid, Spain) and protected from the light for 20 min at RT. The optical densities (OD) were measured at 450 nm with an ELISA reader. The average value of the blanks (wells without serum coating; *N* = 4) was subtracted from all reads. For Piccolo protein quantitation, the human recombinant protein (Abnova, Taipei, Taiwan) was used as standard. Two technical replicates were included for each sample. The Piccolo concentration in serum samples was compared between GBS patients, SCI patients and healthy individuals by Student's t-test with unequal variance (*P* = 0.05).

### Protein-protein interactions and protein ontology annotation

STRING (version 10.0; http://version_10.string-db.org/) was used for the *in silico* characterization of Piccolo-protein interactions [49] using a high confidence interaction score (0.700; http://bit.ly/2anoqgi). Protein ontology analysis for biological process was done using the Blast2GO software (www.blast2go.com).

### Data availability

The iTRAQ mass spectrometry proteomics data are available from the PeptideAtlas available at http://www.peptideatlas.org with identifier PASS00921, or directly via FTP at ftp://PASS00921:NS6844wqa@ftp.peptideatlas.org.

## SUPPLEMENTARY MATERIALS TABLES



## References

[1] Guillain G, Barré JA, Strohl A (1916). Sur un syndrome de radiculonévrite avec hyperalbuminose due liquide céphalo-rachidien sans réaction cellulaire. Remarques sur les caractéres cliniques et graphiques des réflexes tendineux. Bull Soc Méd Hôp Paris.

[2] Van den Berg B, Walgaard C, Drenthen J, Fokke C, Jacobs BC, van Doorn PA (2014). Guillain-Barré syndrome: pathogenesis, diagnosis, treatment and prognosis. Nat Rev Neurol.

[3] Winer JB (2014). An update in Guillain-Barré syndrome. Autoimmune Dis.

[4] Xiao J, Simard AR, Shi FD, Hao J (2014). New strategies in the management of Guillain-Barré syndrome. Clin Rev Allergy Immunol.

[5] Han RK, Cheng YF, Zhou SS, Guo H, He RD, Chi LJ, Zhang LM (2014). Increased circulating Th17 cell populations and elevated CSF osteopontin and IL-17 concentrations in patients with Guillain-Barré syndrome. J Clin Immunol.

[6] Li C, Zhao P, Sun X, Che Y, Jiang Y (2013). Elevated levels of cerebrospinal fluid and plasma interleukin-37 in patients with Guillain-Barré syndrome. Mediators Inflamm.

[7] Li S, Yu M, Li H, Zhang H, Jiang Y (2012). IL-17 and IL-22 in cerebrospinal fluid and plasma are elevated in Guillain-Barré syndrome. Mediators Inflamm.

[8] Wang X, Ma C, Wu J, Zhu J (2013). Roles of T helper 17 cells and interleukin-17 in nueroautoimmune diseases with emphasis on multiple sclerosis and Guillain-Barré syndrome as well as their animal models. J Neurosci Res.

[9] Harness J, McCombe PA (2008). Increased levels of activated T-cells and reduced levels of CD4/CD25+ cells in peripheral blood of Guillain-Barré syndrome patients compared to controls. J Clin Neurosci.

[10] Ang CW, Jacobs BC, Laman JD (2004). The Guillain-Barré syndrome: a true case of molecular mimicry. Trends Immunol.

[11] Kaida K, Ariga T, Yu RK (2009). Antiganglioside antibodies and their pathophysiological effects on Guillain-Barré syndrome and related disorders—a review. Glycobiology.

[12] Rosinska J, Lukasik M, Kozubski W (2012). Neuropathies in the course of primary hepatotropic virus infections. Neurol Neurochir Pol.

[13] Estanislao LB, Morgello S, Simpson DM (2005). Peripheral neuropathies associated with HIV and hepatitis C co-infection: a review. AIDS.

[14] Kuwabara S (2004). Guillain-Barré syndrome: epidemiology, pathophysiology and management. Drugs.

[15] Anaya JM, Ramirez-Santana C, Salgado-Castaneda I, Chang C, Ansari A, Gershwin ME (2016). Zika virus and neurologic autoimmunity: the putative role of gangliosides. BMC Med.

[16] Loshaj-Shala A, Regazzoni L, Daci A, Orioli M, Brezovska K, Panovska AP, Beretta G, Suturkova L (2015). Guillain Barré syndrome (GBS): new insights in the molecular mimicry between C. jejuni and human peripheral nerve (HPN) proteins. J Neuroimmunol.

[17] Sawai S, Satoh M, Mori M, Misawa S, Sogawa K, Kazami T, Ishibashi M, Beppu M, Shibuya K, Ishige T, Sekiguchi Y, Noda K, Sato K, Matsushita K, Kodera Y, Nomura F, Kuwabara S (2014). Moesin is a possible target molecule for cytomegalovirus-related Guillain-Barré syndrome. Neurology.

[18] Doncel-Pérez E, Mateos-Hernández L, Pareja E, García-Forcada A, Villar M, Tobes R, Romero Ganuza F, Vila del Sol V, Ramos R, Fernández de Mera IG, de la Fuente J (2016). Expression of early growth response gene-2 and regulated cytokines correlate with recovery from Guillain-Barré syndrome. J Immunol.

[19] Fiorini M, Zanusso G, Benedetti MD, Righetti PG, Monaco S (2007). Cerebrospinal fluid biomarkers in clinically isolated syndromes and multiple sclerosis. Proteomics Clin Appl.

[20] Yang YR, Liu SL, Qin ZY, Liu FJ, Qin YJ, Bai SM, Chen ZY (2008). Comparative proteomics analysis of cerebrospinal fluid of patients with Guillain-Barré syndrome. Cell Mol Neurobiol.

[21] Ziganshin RH, Ivanova OM, Lomakin YA, Belogurov AA, Kovalchuk SI, Azarkin IV, Arapidi GP, Anikanov NA, Shender VO, Piradov MA, Suponeva NA, Vorobyeva AA, Gabibov AG, Ivanov VT, Govorun VM (2016). The pathogenesis of the demyelinating form of Guillain-Barre syndrome (GBS): proteo-peptidomic and immunological profiling of physiological fluids. Mol Cell Proteomics.

[22] Ziganshin RKh, Arapidi GP, Azarkin IV, Balmasova IP, Timchenko OL, Fed'kina IuA, Morozova EA, Piradov MA, Suponeva NA, Iushchuk ND, Govorun VM (2011). Proteomic technologies for identification of serum potential biomarkers of autoimmune demyelinating polyneuropathies. Bioorg Khim.

[23] Kroksveen AC, Opsahl JA, Aye TT, Ulvik RJ, Berven FS (2011). Proteomics of human cerebrospinal fluid: discovery and verification of biomarker candidates in neurodegenerative diseases using quantitative proteomics. J Proteomics.

[24] Evans RW (1998). Complications of lumbar puncture. Neurol Clin.

[25] Tremlett H, Dai DL, Hollander Z, Kapanen A, Aziz T, Wilson-McManus JE, Tebbutt SJ, Borchers CH, Oger J, Cohen Freue GV (2015). Serum proteomics in multiple sclerosis disease progression. J Proteomics.

[26] Kanda T (2013). Biology of the blood-nerve barrier and its alteration in immune mediated neuropathies. J Neurol Neurosurg Psychiatry.

[27] Hällgren R, Terént A, Venge P (1982). Lactoferrin, lysozyme, and beta 2-microglobulin levels in cerebrospinal fluid: differential indices of CNS inflammation. Inflammation.

[28] Yang YR, Liu SL, Qin ZY, Liu FJ, Qin YJ, Bai SM, Chen ZY (2008). Comparative proteomics analysis of cerebrospinal fluid of patients with Guillain-Barré syndrome. Cell Mol Neurobiol.

[29] Cashman CR, Höke A (2015). Mechanisms of distal axonal degeneration in peripheral neuropathies. Neurosci Lett.

[30] Elf K, Askmark H, Nygren I, Punga AR (2014). Vitamin D deficiency in patients with primary immune-mediated peripheral neuropathies. J Neurol Sci.

[31] D'Aguanno S, Franciotta D, Lupisella S, Barassi A, Pieragostino D, Lugaresi A, Centonze D, D'Eril GM, Bernardini S, Federici G, Urbani A (2010). Protein profiling of Guillain-Barrè syndrome cerebrospinal fluid by two-dimensional electrophoresis and mass spectrometry. Neurosci Lett.

[32] Ellström P, Feodoroff B, Hänninen ML, Rautelin H (2013). Characterization of clinical Campylobacter jejuni isolates with special emphasis on lipooligosaccharide locus class, putative virulence factors and host response. Int J Med Microbiol.

[33] Goodfellow JA, Willison HJ (2016). Antiganglioside, antiganglioside-complex, and antiglycolipid-complex antibodies in immune-mediated neuropathies. Curr Opin Neurol.

[34] Cases-Langhoff C, Voss B, Garner AM, Appeltauer U, Takei K, Kindler S, Veh RW, De Camilli P, Gundelfinger ED, Garner CC (1996). Piccolo, a novel 420 kDa protein associated with the presynaptic cytomatrix. Eur J Cell Biol.

[35] Gundelfinger ED, Reissner C, Garner CC (2016). Role of Bassoon and Piccolo in assembly and molecular organization of the active zone. Front Synaptic Neurosci.

[36] Yamashita T (2011). Glycosphingolipid modification: structural diversity, functional and mechanistic integration of diabetes. Diabetes Metab J.

[37] Nishimune H, Badawi Y, Mori S, Shigemoto K (2016). Dual-color STED microscopy reveals a sandwich structure of Bassoon and Piccolo in active zones of adult and aged mice. Sci Rep.

[38] Kang D, Oh S, Ahn SM, Lee BH, Moon MH (2008). Proteomic analysis of exosomes from human neural stem cells by flow field-flow fractionation and nanoflow liquid chromatography-tandem mass spectrometry. J Proteome Res.

[39] Rozés Salvador V, Heredia F, Berardo A, Palandri A, Wojnacki J, Vivinetto AL, Sheikh KA, Caceres A, Lopez PH (2016). Anti-glycan antibodies halt axon regeneration in a model of Guillain Barrè Syndrome axonal neuropathy by inducing microtubule disorganization via RhoA-ROCK-dependent inactivation of CRMP-2. Exp Neurol.

[40] Rupp A, Morrison I, Barrie JA, Halstead SK, Townson KH, Greenshields KN, Willison HJ (2012). Motor nerve terminal destruction and regeneration following anti-ganglioside antibody and complement-mediated injury: an in and ex vivo imaging study in the mouse. Exp Neurol.

[41] Shin T, Ahn M, Matsumoto Y, Moon C (2013). Mechanism of experimental autoimmune neuritis in Lewis rats: the dual role of macrophages. Histol Histopathol.

[42] Shevchenko A, Tomas H, Havlis J, Olsen JV, Mann M (2006). In-gel digestion for mass spectrometric characterization of proteins and proteomes. Nat Protoc.

[43] Köcher T, Pichler P, Schutzbier M, Stingl C, Kaul A, Teucher N, Hasenfuss G, Penninger JM, Mechtler K (2009). High precision quantitative proteomics using iTRAQ on an LTQ Orbitrap: a new mass spectrometric method combining the benefits of all. J Proteome Res.

[44] Martinez-Bartolome S, Navarro P, Martin-Maroto F, Lopez-Ferrer D, Ramos-Fernandez A, Villar M, Garcia-Ruiz JP, Vazquez J (2008). Properties of average score distributions of SEQUEST the probability ratio method. Mol Cell Proteomics.

[45] Navarro P, Vazquez J (2009). A refined method to calculate false discovery rates for peptide identification using decoy databases. J Proteome Res.

[46] Bonzon-Kulichenko E, Garcia-Marques F, Trevisan-Herraz M, Vazquez J (2015). Revisiting peptide identification by high-accuracy mass spectrometry problems associated with the use of narrow mass precursor windows. J Proteome Res.

[47] Navarro P, Trevisan-Herraz M, Bonzon-Kulichenko E, Nunez E, Martinez-Acedo P, Perez-Hernandez D, Jorge I, Mesa R, Calvo E, Carrascal M, Hernaez ML, Garcia F, Barcena JA, Ashman K, Abian J, Gil C, Redondo JM, Vazquez J (2014). General statistical framework for quantitative proteomics by stable isotope labeling. J Proteome Res.

[48] García-Marqués F, Trevisan-Herraz M, Martínez-Martínez S, Camafeita E, Jorge I, Lopez JA, Méndez-Barbero N, Méndez-Ferrer S, del Pozo MA, Ibáñez B, Andrés V, Sánchez-Madrid F, Redondo JM, Bonzon-Kulichenko E, Vázquez J (2016). A novel systems-biology algorithm for the analysis of coordinated protein responses using quantitative proteomics. Mol Cell Proteomics.

[49] Szklarczyk D, Franceschini A, Wyder S, Forslund K, Heller D, Huerta-Cepas J, Simonovic M, Roth A, Santos A, Tsafou KP, Kuhn M, Bork P, Jensen LJ, von Mering C (2015). STRING v10: protein-protein interaction networks, integrated over the tree of life. Nucleic Acids Res.

